# Customizing the Emergence Profile Around an Immediately Loaded Single Implant in the Esthetic Zone: A Case Report

**DOI:** 10.7759/cureus.58279

**Published:** 2024-04-15

**Authors:** Grazina Fernandes, Ashwin Mysore, Omkar Shetye, Meena Aras, Vidya Chitre

**Affiliations:** 1 Department of Prosthodontics and Crown and Bridge, Goa Dental College and Hospital, Bambolim, IND; 2 Department of Oral and Maxillofacial Surgery, Goa Dental College and Hospital, Bambolim, IND

**Keywords:** customizing soft tissue, ridge augmentation, prosthetic rehabilitation, gingival recontouring, soft tissue contour, interim restoration, immediate loading, esthetics, implants, emergence profile

## Abstract

An optimal esthetic result is essential for an implant-supported restoration in the anterior zone. In the esthetic zone, providing immediate interim restorations following implant surgery has been proposed as a reliable and desirable treatment approach. A well-contoured interim restoration following implant placement minimizes hard and soft tissue changes in the peri-implant zone. This in turn has the potential to enhance the esthetic outcome and, therefore, patient satisfaction. Multiple prosthetic and surgical aspects need to be carefully planned and executed to achieve the intended final result. This is a report describing the steps involved in recontouring the gingiva to achieve an optimal emergence profile following the immediate loading of a single implant in the esthetic zone.

## Introduction

Implant therapy is currently regarded as the most suitable method for replacing missing teeth, particularly in the anterior zone. The essential requirement for the success of implants is osteointegration. One of the most important elements in ensuring the longevity of implants and determining the ideal hard and soft tissues is the emergence profile. The emergence profile of dental implant restorations should resemble natural teeth. Inflamed soft tissue and limited access to oral hygiene are consequences of improperly shaped tissues and restorations that can lead to unappealing outcomes [1,2]. For both esthetic and functional implant therapy, a well-contoured restoration with a natural emergence profile and gingival structure that blends in with surrounding teeth is crucial [3].

In 1977, Stein and Kuwata introduced the term "emergence profile" to describe the contours of teeth and crowns as they traversed soft tissue and ascended toward the interproximal contact area and the buccal and lingual height of contour [4]. According to Croll's photographic analysis of natural teeth, emergence profiles are generally straight rather than convex or concave [5]. To provide favorable conditions for oral hygiene, interim crowns can be modified gradually by guided conditioning employing a dynamic compression technique in the peri-implant area [6].

This case report describes a technique to fabricate a screw-retained interim restoration using composite resin for immediate loading followed by transfer of the emergence profile using a customized impression coping.

## Case presentation

**Figure 1:**
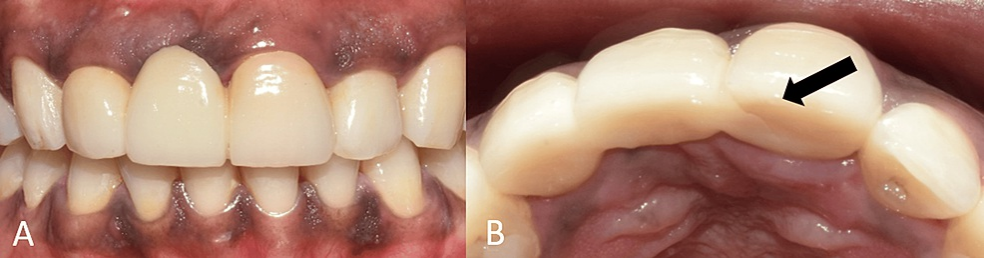
Pre-operative intraoral view showing fractured FPD. (A) Frontal view on maximum intercuspation showing the FPD in relation to teeth no. 11, 12, and 21; (B) occlusal view showing fractured FDP on the palatal aspect (black arrow). FPD, fixed partial denture

**Figure 2:**
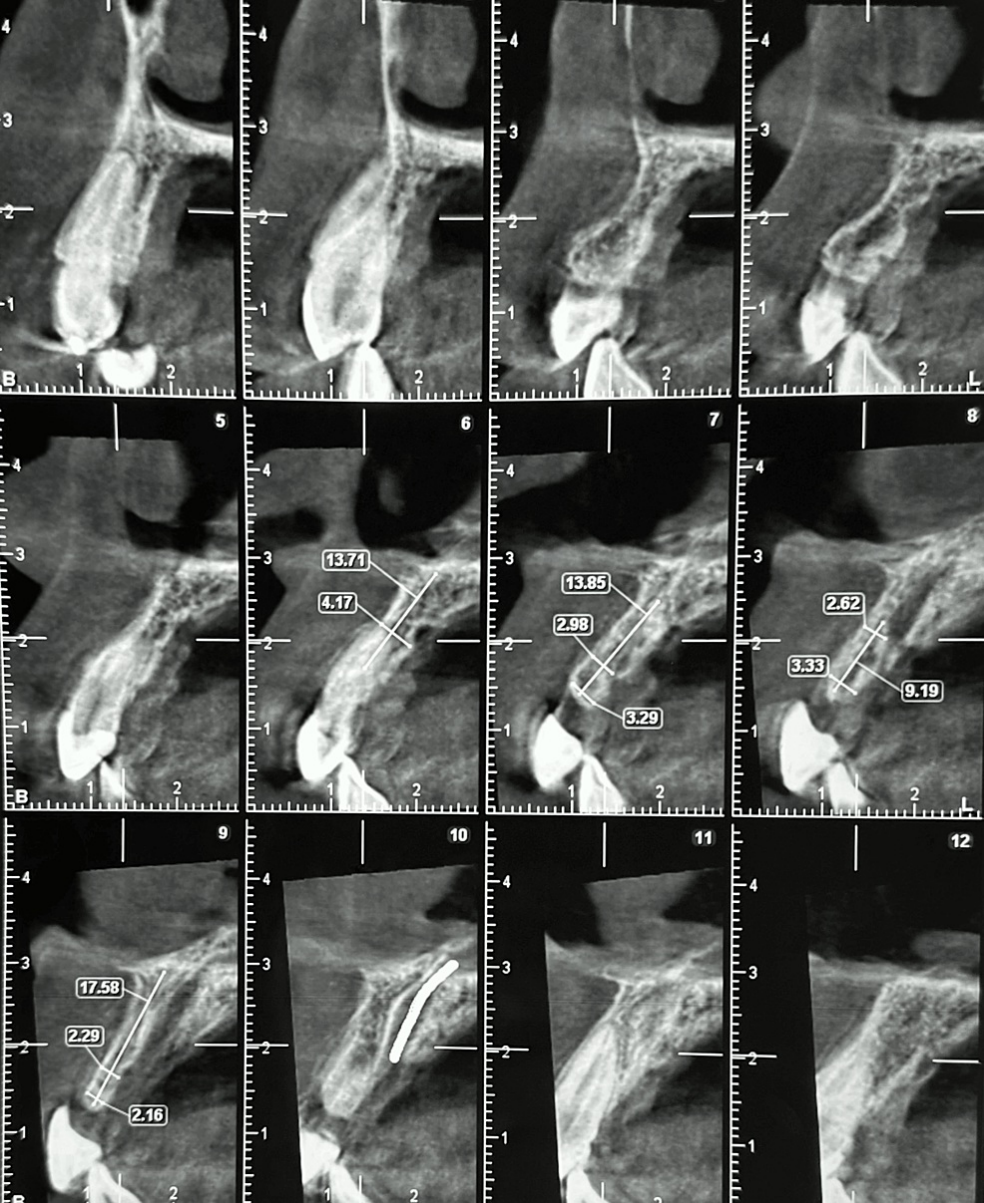
Pre-operative CBCT of anterior maxilla showing bone width at different sections. CBCT, cone beam computed tomography

A healthy 25-year-old female patient presented with a fractured fixed partial denture (FPD) of teeth 11, 12, and 21. Clinical examination revealed a fracture of the pontic on the palatal aspect (Figure 1). A cone beam computed tomography (CBCT) scan was performed, which revealed horizontal alveolar bone resorption at the missing central incisor site (Figure 2). Rehabilitation of the missing incisor with a dental implant following bone augmentation was decided upon as the treatment of choice.

**Figure 3:**
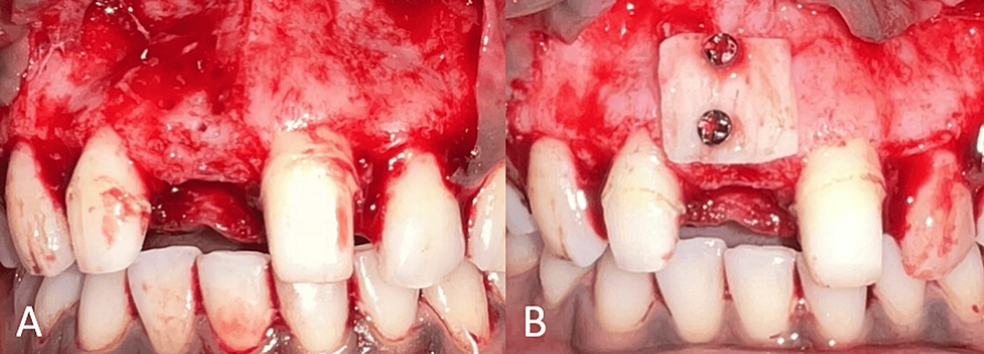
Surgical procedure. (A) Flap elevation showing the labial defect; (B) placement of autogenous bone block harvested from the mandibular symphysis.

Following the removal of the FPD, the patient was instructed to rinse with 0.2% chlorhexidine for one minute before beginning the surgical procedure. The perioral skin was disinfected with a 5% povidone-iodine solution. Local anesthetic (2% lignocaine with 1:80,000 adrenaline) was used during the surgery. In addition to releasing incisions, crestal and intrasulcular incisions were made on the labial surfaces of adjacent teeth to obtain access. A mucoperiosteal flap of full thickness was raised (Figure 3A).

**Figure 4:**
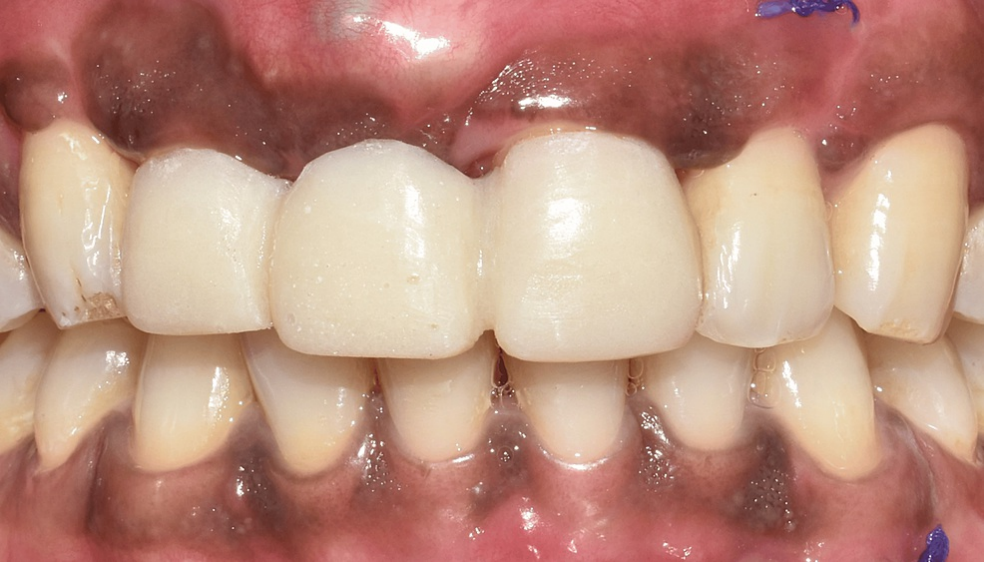
Frontal view showing heat-cured acrylic FPD in relation to tooth no. 11, 12, and 21. FPD, fixed partial denture

The Khoury technique of bone augmentation was used; wherein an autogenous bone block was harvested from the symphysis region of the mandible and stabilized using two microscrews (1.5x10 mm, AK instruments, India) on the labial aspect of the defect (Figure 3B). The generated gap was filled with a combination of autogenous and deproteinized bovine bone xenograft (Bio-Oss; small granules, Geistlich Pharma, Wolhusen, Switzerland). The flap was sutured and primary closure was obtained. Postoperative instructions were provided to the patient. A heat-cured acrylic FPD was cemented post-operatively to serve as a tooth-supported provisional restoration (Figure 4).

**Figure 5:**
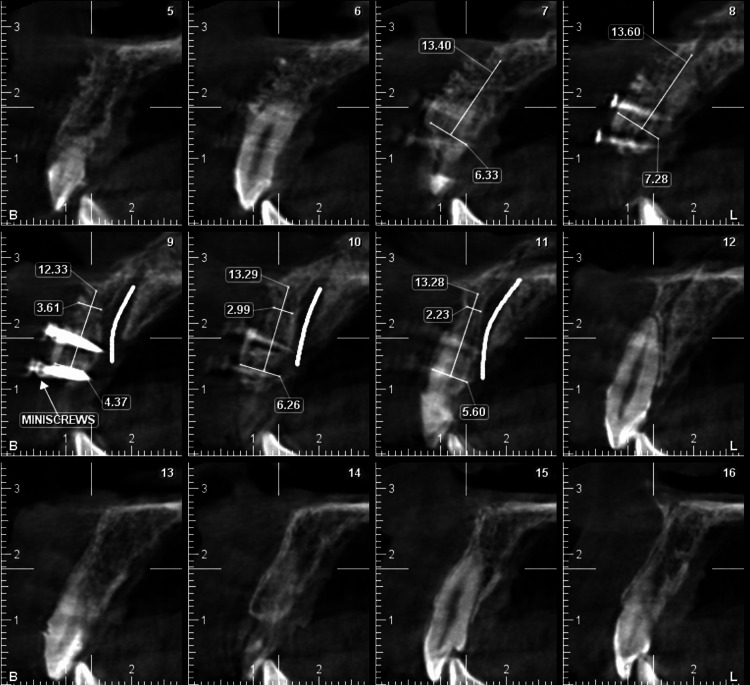
Post-operative CBCT of anterior maxilla showing the regenerated site and microscrews at different sections. CBCT, cone beam computed tomography

**Figure 6:**
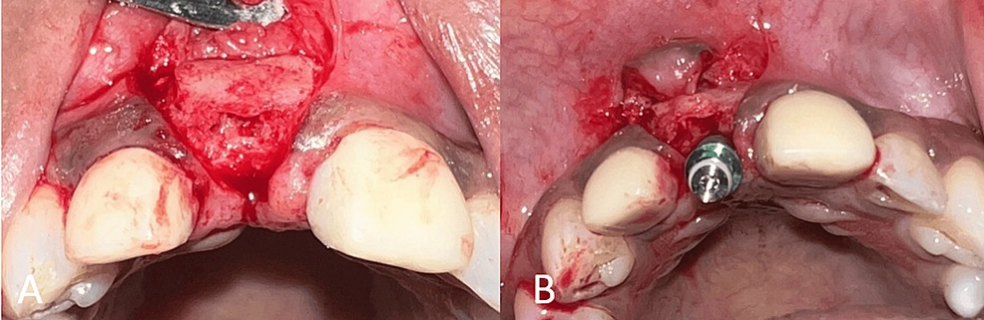
Surgical procedure. (A) Flap elevation showing the regenerated site; (B) implant placement in relation to tooth no. 11.

After six months of healing, the site was reopened using a modified papilla-sparing incision, and a full-thickness mucoperiosteal flap was raised for implant placement. The bone demonstrated a horizontal width of 6 mm (Figure 5). The two microscrews were removed and a 4x11.5 mm implant (Osstem implant TS III, Seoul, Korea) was placed in the central incisor region (Figure 6).

**Figure 7:**
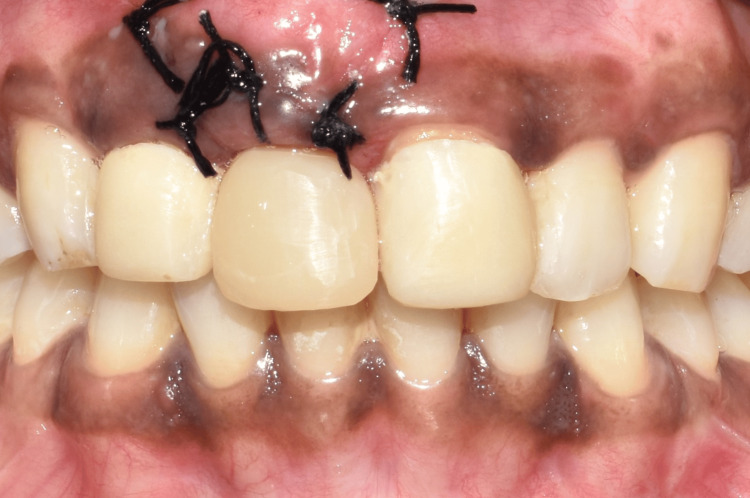
Screw-retained interim implant restoration.

The temporary abutment was positioned at the surgical site following the implant insertion. To fabricate an interim restoration, the composite resin was incrementally built up over and around the temporary abutment (Figure 7).

**Figure 8:**
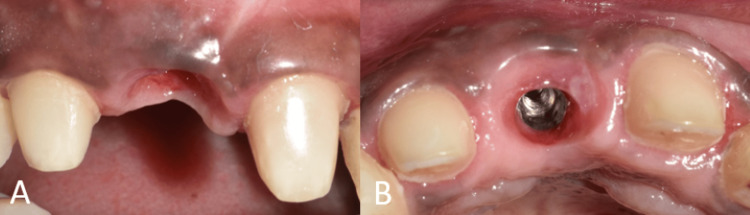
Emergence profile after customization. (A) Frontal view showing the customized emergence profile in relation to tooth no. 11; (B) occlusal view showing the customized emergence profile in relation to tooth no. 11.

To obtain the desired emergence profile, the soft tissue around the interim restoration was molded by relining the composite every two weeks for a period of two months (Figure 8).

**Figure 9:**
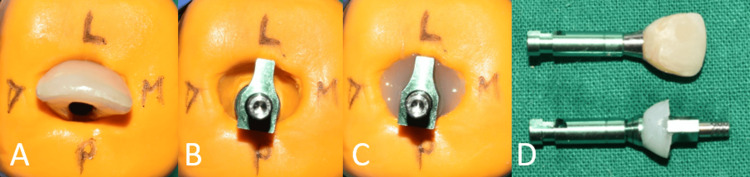
Customizing the open-tray impression coping. (A) Placement of the interim restoration in the putty index; (B) removal of interim restoration followed by placement of the open-tray impression coping in the putty index; (C) placement of flowable composite into the space between putty and the impression coping; (D) contours of the interim restoration transferred to the impression coping.

**Figure 10:**
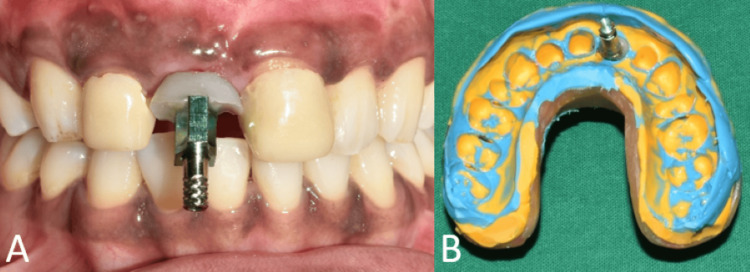
Open-tray implant impression to fabricate the prosthesis. (A) Frontal view showing the customized impression coping; (B) open-tray implant impression.

A final implant prosthesis was fabricated after the desired emergence profile was achieved. The gingival contour that was achieved using the prior restoration was transferred to an open-tray impression coping using the technique recommended by Hinds, following which an implant-level impression was made (Figure 9 and Figure 10) [7].

**Figure 11:**
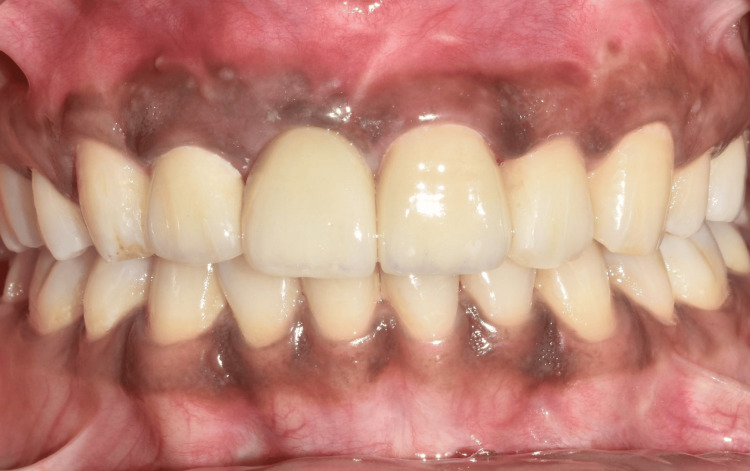
Frontal view showing the final implant prosthesis.

**Figure 12:**
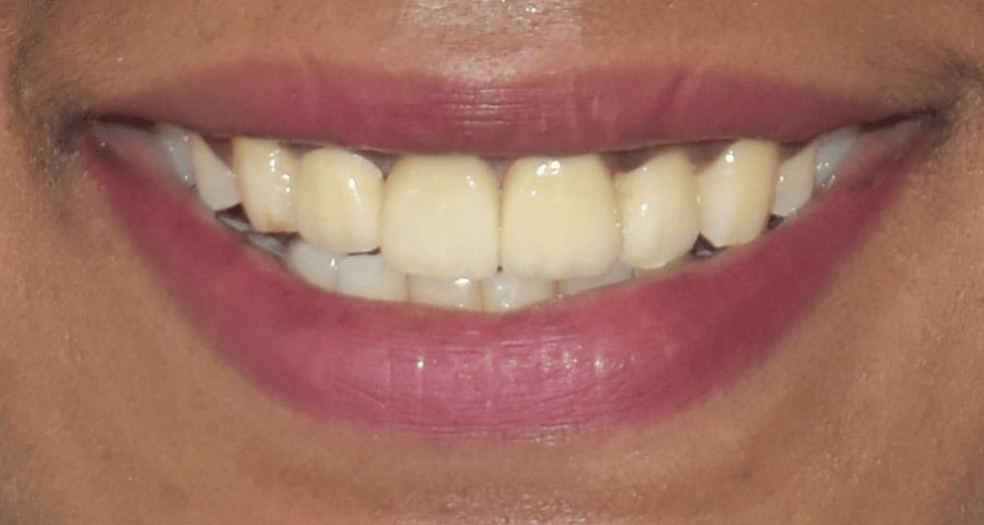
Extraoral picture of the patient smiling after final restorations.

The definitive prosthesis was fabricated and inserted (Figure 11 and Figure 12).

## Discussion

In the maxillary anterior area, achieving optimal esthetic outcomes with dental implants is difficult and depends on bone quantity, quality, and interdental papilla preservation [8]. Benefits of immediately loaded implants include immediate function, interim restoration insertion without the need for second-stage surgery, a reduction in treatment time and trauma, and psychological benefits [9].

A favorable emergence profile supports the soft tissue around restorations; it allows the maintenance of oral hygiene [10]. For implant-supported restorations, a provisional restoration can aid in both obtaining and planning the desired esthetic outcome. A significant benefit of temporary restorations is the ability to contour the gingival architecture as it heals [11]. Various methods have been suggested to restore gingival contour, establish an emergence profile, and transfer the contour for an esthetic final restoration. Neale and Chee recommended recontouring the tissues using gingivoplasty techniques before fabricating interim restoration [1]. Ormianer et al. transferred the soft tissue shape by immediately placing autopolymerizing acrylic resin into the sulcus [12]. However, intraoral usage of acrylic resin monomer may irritate soft tissue chemically and thermally. Hinds et al. described a technique to customize impression coping for replicating the healed soft tissue [7].

The technique described in this article minimizes gingival discomfort by preventing the intraoral use of resin monomer. By reshaping the soft tissue as required throughout the healing process, it also reduces the need for second-stage surgery. A further significant benefit is the prevention of soft tissue collapse during the impression process, since the impression copings that are customized provide an accurate transfer of the peri-implant soft tissue contours to the cast and, therefore, to the final restorations.

## Conclusions

In the esthetic zone, careful patient selection, diagnosis, and treatment planning are necessary to prevent undesirable results. Consideration must be given to the parameters at each stage, from implant placement to the postoperative phase, to create a harmonious and esthetic emerging profile. An implant prosthesis needs to have both a functional and esthetically pleasing design. The process of gingival recontouring with a temporary restoration is non-invasive. By adjusting the emerging profile during the healing period, it is possible to effectively achieve the final result, which consists of a soft tissue structure that is harmonious with the contours of the restoration.
